# Mobile phones and malignant melanoma of the eye

**DOI:** 10.1038/sj.bjc.6600068

**Published:** 2002-02-01

**Authors:** C Johansen, J D Boice, J K McLaughlin, H C Christensen, J H Olsen

**Affiliations:** Danish Cancer Society, Institute of Cancer Epidemiology, Strandboulevarden 49, DK-2100 Copenhagen Ø, Denmark; International Epidemiology Institute, 1455 Research Blvd, Suite 550, Rockville, Maryland, MD 20850, USA; Department of Medicine, Vanderbilt Medical Center and Vanderbilt-Ingram Comprehensive Cancer Center, Nashville, Tennessee, TN 37232, USA

**Keywords:** cellular telephones, ocular melanoma, epidemiology

## Abstract

Recently a four-fold increase in the risk of malignant melanoma of the eye was associated with the use of radiofrequency transmitting devices, including mobile phones in Germany. We contrasted the incidence rates of this rare cancer with the number of mobile phone subscribers in Denmark. We observed no increasing trend in the incidence rate of melanoma, which was in sharp contrast to the exponentially increasing number of mobile phone subscribers starting in the early 1980s. Our study provides no support for an association between mobile phones and ocular melanoma.

*British Journal of Cancer* (2002) **86**, 348–349. DOI: 10.1038/sj/bjc/6600068 www.bjcancer.com

© 2002 The Cancer Research Campaign

## 

The widespread use of cellular telephones throughout the world has raised concern about possible adverse health effects. Recent studies have failed to reveal a link between brain cancer and use of cellular telephones (Muscat *et al*, 2000; Inskip *et al*, 2001; Johansen *et al*, 2001), although there were limitations in terms of the duration of phone use and length of follow-up. A recently published case–control interview study from Germany reported a significant four-fold increased risk of malignant melanoma of the eye associated with the use of radiofrequency (RF) transmitting devices, including mobile telephones (Stang *et al*, 2001). These data were generally consistent with a previous case–control investigation linking uveal melanoma with occupations involving radiofrequency exposures to radar and microwaves (Holly *et al*, 1996). To evaluate whether radiofrequency exposure might increase the risk of ocular cancer, we compared incidence data from the Danish Cancer Registry with information on the number of cellular telephone users in Denmark over the past two decades.

## MATERIALS AND METHODS

Our analysis included all ocular malignant melanomas identified from 1943 through 1996 by the Danish Cancer Registry, classified according to the modified Danish version of *The International Classification of Diseases, 7th Revision* (ICD-7). The age-specific incidence rates were calculated using 5-year age intervals and 5-year period intervals. Incidence rates were age-standardized to the World Standard Population (WSP). Data on subscribers to mobile telephones in Denmark from 1982 through 1996 were obtained from the National Board of Telecommunication.

## RESULTS

During the period 1943 through 1996, the mean number of incident cases of ocular malignant melanoma in each 5-year calendar period increased from approximately 30 to 60 (Table 1

**Table 1:**
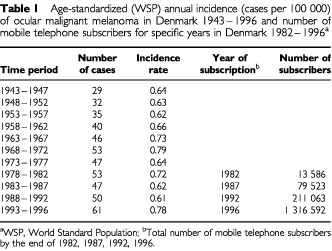
Age-standardized (WSP) annual incidence (cases per 100 000) of ocular malignant melanoma in Denmark 1943–1996 and number of mobile telephone subscribers for specific years in Denmark 1982–1996^a^

). However, the age-standardized incidence rates remained fairly stable with only small and irregular changes between 0.61 and 0.79 per 100 000 per year, consistent with values reported in other countries (Sahel *et al*, 2001). The absence of a rising trend in incidence is seen in Figure 1

**Figure 1:**
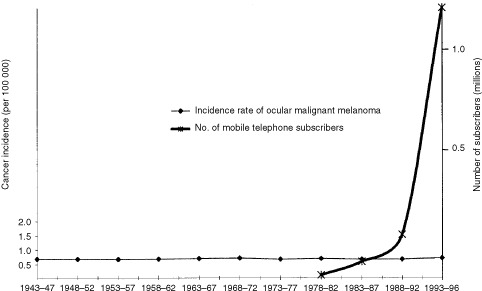
Age-standardized World Standard Population (WSP) annual incidence (cases per 100 000) of ocular malignant melanoma in Denmark 1943–1996 and number of subscribers to cellular telephones, Denmark 1982–1996 (WSP).

and contrasted with the exponential increase in the number of subscribers to mobile telephones in Denmark from 1982 to 1996.

## DISCUSSION

The findings of this descriptive epidemiological study do not support an association between use of mobile telephones and risk of ocular malignant melanoma. The long-term relatively stable incidence rate suggests that the risk factors for this rare cancer, such as dysplastic nevus syndrome, atypical ocular nevi, iris pigmentation and ocular and oculardermal melanocytosis (Sahel *et al*, 2001) have changed little over the 54 years of incidence data available in Denmark.

In addition to the absence of an increasing trend in incidence of melanoma of the eye, we found no association between this cancer and cellular phone use in our ongoing cohort study of over 420 000 users of mobile telephones between 1982 and 1995 (Johansen *et al*, 2001). Eight cases of ocular cancer were observed compared with 13.5 cases expected (standardized incidence ratio, 0.59; 95% confidence interval, 0.25–1.17).

Conceivably, the period of use of cellular telephones in Denmark may be too brief to detect an early-stage effect of RF. However, because RF signals from cellular phones are unlikely to break chemical bonds or cause gene mutations, the biologic process underlying a possible mechanism would be one that promotes tumour growth (Repacholi, 1997), although experimental data supporting a promotional mechanism of RF are scant (National Radiological Protection Board, 2000). If RF exposure is assumed to act by promoting the growth of an underlying ocular lesion, then the large number of recent cellular phone users in Denmark should be sufficient to detect such an effect.

It is not entirely clear why results from our descriptive and cohort studies on cellular telephone use and uveal melanoma differ so strikingly from the German case-control study (Stang *et al*, 2001), but reasons might include response and other biases usually associated with interview case–control studies (Wacholder *et al*, 1992) or the inability to adjust for ultraviolet light or other potentially important confounding factors (Inskip, 2001). Nonetheless, the results of our analyses do not support the hypothesis that malignant melanoma of the eye is a consequence of exposure to radiofrequency waves from mobile phones, although the incidence rate for the most recent period suggests that further data are necessary to settle the issue.

Although the exposure to the eyes from RF from mobile phones is low, any future analytic studies might benefit from refined cellular telephone exposure assessment that would enable risk evaluation over categories of exposure. Likewise, inclusion of information on possible confounders such as exposure to ultraviolet radiation and predisposing medical conditions mentioned above should be considered.
